# Monoclonal Antibodies Opsonize *Burkholderia* spp. and Reduce Intracellular Actin Tail Formation in a Macrophage Infection Assay

**DOI:** 10.1128/JB.00244-21

**Published:** 2021-10-12

**Authors:** A. Taylor, D. Jenner, C. Rowland, T. Laws, I. Norville, J. Prior

**Affiliations:** a Defence Science and Technology Laboratory, Chemical, Biological and Radiological Division, Salisbury, United Kingdom; b London School of Hygiene and Tropical Medicine (LSHTM), London, United Kingdom; c University of Exeter, Exeter, United Kingdom; d University of Southampton, Southampton, United Kingdom; Brigham and Women’s Hospital/Harvard Medical School

**Keywords:** actin, antibody, macrophage, melioidosis, opsonization

## Abstract

Melioidosis is a neglected tropical disease caused by the bacterium Burkholderia pseudomallei. The bacterium is intrinsically resistant to various antibiotics, and melioidosis is therefore difficult to treat successfully without a relapse in infection. B. pseudomallei is an intracellular pathogen and therefore, to eradicate the infection, antimicrobials must be able to access bacteria in an intracellular niche. This study assessed the ability of a panel of monoclonal antibodies (MAbs) to opsonize Burkholderia species and determine the effect that each antibody has on bacterial virulence *in vitro*. Murine macrophage infection assays demonstrated that monoclonal antibodies against the capsule of B. pseudomallei are opsonizing. Furthermore, one of these monoclonal antibodies reduced bacterial actin tail formation in our *in vitro* assays, indicating that antibodies could reduce the intracellular spread of Burkholderia thailandensis. The data presented in this paper demonstrate that monoclonal antibodies are opsonizing and can decrease bacterial actin tail formation, thus decreasing their intracellular spread. These data have informed selection of an antibody for development of an antibody-antibiotic conjugate (AAC) for melioidosis.

**IMPORTANCE** Melioidosis is difficult to treat successfully due to the causal bacterium being resistant to many classes of antibiotics, therefore limiting available therapeutic options. New and improved therapies are urgently required to treat this disease. Here, we have investigated the potential of monoclonal antibodies to target this intracellular pathogen. We have demonstrated that monoclonal antibodies can target the bacterium, increase uptake into macrophages, and reduce actin tail formation required by the bacterium for spread between cells. Through targeting the bacterium with antibodies, we hope to disarm the pathogen, reducing the spread of infection. Ultimately, we aim to use an opsonizing antibody to deliver antibiotics intracellularly by developing an antibody-antibiotic conjugate therapeutic for melioidosis.

## INTRODUCTION

Melioidosis is a potentially fatal neglected tropical disease caused by the Gram-negative bacterium Burkholderia pseudomallei. It has been estimated that 165,000 cases of melioidosis occur worldwide each year, with up to 89,000 deaths (1). Melioidosis is often misdiagnosed due to the wide variety of symptoms associated with the disease (2, 3). Even with successful diagnosis and treatment, studies have shown relapse rates of between 13 and 23% in survivors (4–7), with the majority of cases occurring within 12 months of initial therapy. Due to the complex antibiotic therapy, rates of relapse and the prevalence of antimicrobial resistance, it is clear that new therapeutic approaches are required to aid in the treatment of melioidosis. Monoclonal antibody (MAb) therapies have been in development in the oncology field for many years (8), with research and development into antibody-based therapeutics for infectious diseases accelerating in recent years (9).

B. pseudomallei is well equipped to evade killing by the immune system. One aspect to this is its ability to reside within host cells, therefore avoiding detection and denying access to humoral defenses. The bacterium has a large genome of 7.2 Mb, which is split between two chromosomes. These chromosomes encode virulence factors such as capsular polysaccharide (CPS), lipopolysaccharide (LPS), adhesins, flagella, and secretory systems, of which the type three and type six secretory systems are required for optimum intracellular survival and virulence (10–14).

B. pseudomallei can infect a variety of phagocytic and nonphagocytic cells. B. pseudomallei adhesins, such as BoaA and BoaB, are important factors for adhesion to nonphagocytic cells (14). Following phagocytosis into macrophage cells, B. pseudomallei can escape endocytic vacuoles using the type three secretory system (T3SS) apparatus (15, 16). Once in the cytosol, the bacteria move via actin-based motility; this process is a bacterially mediated process that involves the bacteria recruiting host actin from the cytoskeleton in the form of G actin, when is then assembled into F actin by mimicking host cell nucleation-promoting factors (17–21). In B. pseudomallei and Burkholderia thailandensis, this process is driven by the BimA gene. Although the type VI secretion systems of B. thailandensis and B. pseudomallei are responsible for the cellular fusion, the motility of the bacterium is an important aspect of mobility to facilitate spread (18). Actin-based motility is not unique to B. thailandensis, B. pseudomallei, and B. mallei; for example, bacteria from the Shigella and Listeria genus can also utilize actin for intracellular motility (22, 23). It is thought that actin-based motility leads to multinucleated giant cell (MNGC) formation via the force exhibited on the cell membrane promoting contact with adjacent cells (24). For B. pseudomallei and B. thailandensis, MNGC formation is an important aspect of cellular invasion that can be seen in both phagocytic and nonphagocytic cells, where intracellular bacterial replication eventually leads to cell damage and plaque formation (25). The ability of B. pseudomallei to survive intracellularly, thus avoiding some traditional antibiotic therapies, highlights the importance of investigating novel antimicrobial therapies for melioidosis.

Burkholderia thailandensis shares many similarities to the hazard group 3 pathogen B. pseudomallei, although B. thailandensis is rarely pathogenic to humans and is therefore a hazard group 2 organism. B. thailandensis E555 can be used as a surrogate for B. pseudomallei, as the bacterium possesses a polysaccharide capsule identical to the polysaccharide capsule of B. pseudomallei (26) and is virulent in macrophage cell culture infection assays. Techniques for analyzing intracellular bacteria within RAW 264.7 macrophages by CFU and imaging flow cytometry have been previously reported for B. thailandensis (27). A range of anti-*Burkholderia* murine monoclonal antibodies developed and owned by the Defence Science and Technology Laboratory (Dstl) have previously been tested *in vivo* for protection against B. pseudomallei in mice (28), with the capsule-specific antibodies demonstrating a level of protection.

In this study, we test a range of *Burkholderia*-specific monoclonal antibodies for their abilities to interfere with B. pseudomallei and B. thailandensis *in vitro* infection and, as such, their suitability for use as therapies for melioidosis. The aim is to investigate opsonization and its effect on intracellular bacteria. These antibody data set the foundations for the generation of a proof-of-principle antibody-antibiotic conjugate (AAC) for melioidosis.

## RESULTS

### Monoclonal antibodies are opsonizing in a RAW 264.7 macrophage infection assay.

A RAW 264.7 macrophage infection assay was used to determine monoclonal antibody opsonization. Opsonization was assessed by measuring bacterial uptake with a viable count assay and imaging flow cytometry, initially using B. thailandensis E555. Each MAb (Table 1) was added to B. thailandensis concentrations ranging from 0.1 ng · ml^−1^ to 1 µg · ml^−1^ (anti-CPS MAbs 4VIH12, 3VIE5, and 4VA5) and from 10 ng · ml^−1^ to 100 µg · ml^−1^ (anti-LPS MAb CC6). Antibodies were incubated for 30 min with the B. thailandensis cells to allow binding prior to macrophage infection. Numbers of viable bacteria (CFU/well) were determined by lysis of intact macrophage cells and enumeration of the intracellular bacteria by serial dilution and culture on Luria agar plates. Imaging flow cytometry of intact macrophage cells was used as an additional method to determine antibody opsonization, together with confocal microscopy for high-definition imaging of intracellular bacterial infection.

**TABLE 1:** Monoclonal antibodies evaluated in this study

Antibody	Target^*a*^	Isotype	Species
4VIH12	Anti-CPS	IgG2b	Murine
3VIE5	Anti-CPS	IgG2b	Murine
4VA5	Anti-CPS	IgG1	Murine
CC6	Anti-LPS	IgG2a	Murine

a CPS, capsular polysaccharide; LPS, lipopolysaccharide.

**FIG 1:**
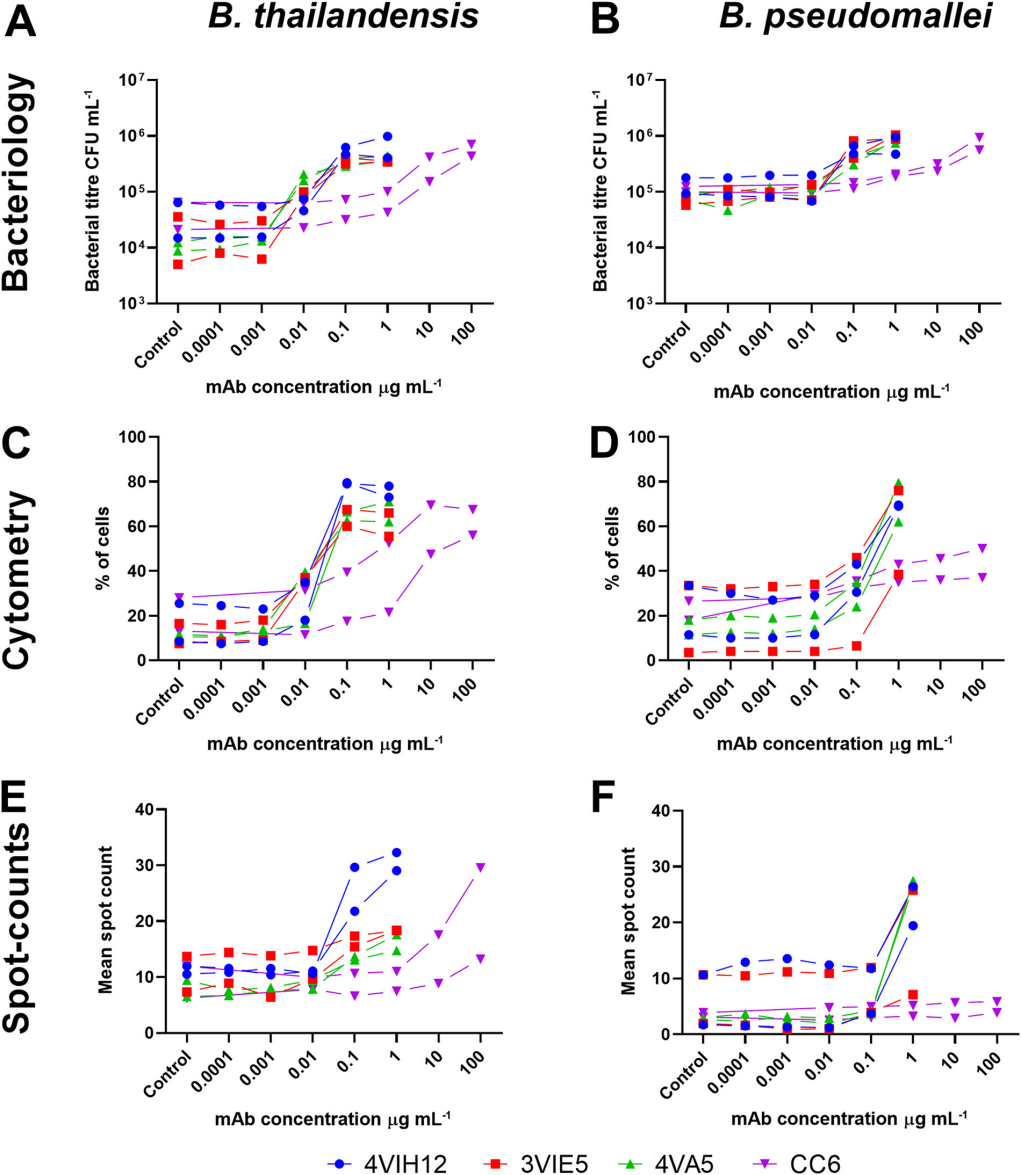
Monoclonal antibody opsonization. Burkholderia thailandensis (left) and Burkholderia pseudomallei (right) were opsonized at a range of concentrations of anti-capsular polysaccharide (CPS) monoclonal antibodies (MAbs) (4VIH12, 3VIE5, and 4VA5) and anti-lipopolysaccharide (LPS) MAb (CC6; note higher concentrations). Intracellular bacteria within RAW 264.7 macrophages were analyzed ay 2 h postinfection by CFU enumeration (A, B), imaging flow cytometry (C, D), and spot counts (E, F). For each concentration of MAb, replicates were performed across two separate experimental days. Controls consisted of an isotype control antibody at the highest concentration. (A to D) Statistics for bacteriology and cytometry. *P* < 0.001 (analysis of covariance [ANCOVA]) for MAb dose response); *P* < 0.001 for all Bonferroni’s posttests in which CC6 MAb was compared to each of the other MAbs. (E, F) Statistics for spot counts. *P* < 0.001 (ANCOVA for MAb dose response). Imaging flow cytometry gating and spot count analysis are shown in Fig. S1 in the supplemental material.

Intracellular CFU enumeration experiments showed that each MAb analyzed was opsonizing toward B. thailandensis E555 in the RAW264.7 macrophage infection assay (Fig. 1A). A statistical modeling approach was taken to analyze the data, where the logarithmic transformation of dose was included as a covariate and the bacteria, antibody, and replicate experiments were included as fixed factors and with all interactions. Each anti-CPS MAb is opsonizing in a dose-dependent manner (*P* < 0.001) by increasing intracellular CFU level by a greater than 1-log fold increase. An average CFU of 2.1 × 10^4^ CFU · ml^−1^ is observed at 0.0001 µg · ml^−1^ of anti-CPS MAb; this increases to 4 × 10^5^ CFU · ml^−1^ at an anti-CPS MAb concentration of 0.1 µg · ml^−1^. In comparison, an anti-LPS antibody concentration of 10 µg · ml^−1^ or greater is required to achieve a similar level of intracellular CFU increase. Modeling suggested differences between antibodies (*P* < 0.001) with a greater concentration of the anti-LPS MAb CC6 required to achieve an increase in B. thailandensis intracellular CFU compared to the anti-CPS MAbs 4VIH12, 3VIE5, and 4VA5 (*P* < 0.001, for all Bonferroni’s posttests in which CC6 MAb was compared to each of the other MAbs).

All MAbs analyzed for opsonization by imaging flow cytometry showed a dose-dependent ability to increase the percentage of cells infected with B. thailandensis (Fig. 1C). A similar modeling approach was taken as with the CFU data. Again, there was a dose response for the antibodies (*P* < 0.001) and a clear difference between the anti-CPS MAbs (*P* < 0.001) (4VIH12, 3VIE5, and 4VA5) and the anti-LPS MAb (CC6), mirroring the result seen by intracellular CFU analysis (*P* < 0.001 for all Bonferroni’s posttests in which CC6 MAb was compared to each of the other MAbs). Additionally, imaging flow cytometry can be used to estimate the proportional extent of bacterial presence within the cell by “spot counting” in the focal plane of each cell (see Fig. S1 in the supplemental material). Although the spot counting profiles (Fig. 1E) differ between MAbs and bacterial strains, the data indicate an increase in the average number of intracellular bacteria per cell when opsonized with MAb. The spot count analysis broadly demonstrated the same conclusions as the CFU-associated data, demonstrating a dose effect (*P* < 0.001; analysis of covariance [ANCOVA] for dose response).

### The ability of monoclonal antibodies to opsonize B. pseudomallei K96243 is comparable to ability to opsonize B. thailandensis E555.

The MAb panel was assessed to determine if the opsonization ability demonstrated for B. thailandensis E555 can also be applied to the highly pathogenic (Advisory Committee on Dangerous Pathogens, UK (ACDP, level III B. pseudomallei K96243 (Fig. 1B, D, and F). A red fluorescent protein (RFP)-expressing strain of B. pseudomallei K96243 was used to enable visualization of the bacterium within macrophage cells by imaging flow cytometry.

Analysis of intracellular bacteria by CFU enumeration and imaging flow cytometry showed that opsonization of B. pseudomallei K96243 by each MAb is comparable to the data for that of B. thailandensis E555 (Fig. 1). These data were analyzed within the modeling discussed in the previous section, alongside the B. thailandensis data. We found overall levels of uptake were different when measuring CFU (*P* < 0.001) or flow cytometry (*P* = 0.009). However, these differences did not seem to alter the role of antibody when measuring CFU (*P* < 0.810) or flow cytometry (*P* = 0.891). The conclusions are therefore the same as those with B. thailandensis. Each antibody was opsonizing and significantly increased bacterial uptake with dose (*P* < 0.001; ANCOVA for dose response); anti-CPS MAbs (4VIH12, 3VIE5, and 4VA5) outperformed the anti-LPS MAb (CC6) in terms of opsonization ability (*P* < 0.001 for all Bonferroni’s posttests in which CC6 MAb was compared to each of the other MAbs). Antibody opsonization data were used to select an anti-CPS antibody for further analysis in cell infection assays. The anti-CPS MAb 3VIE5 was selected because this MAb had previously been shown in other studies to have favorable binding kinetics (data not shown).

### Opsonized B. thailandensis bacteria have a reduction in actin tail formation within RAW 264.7 macrophages.

**FIG 2:**
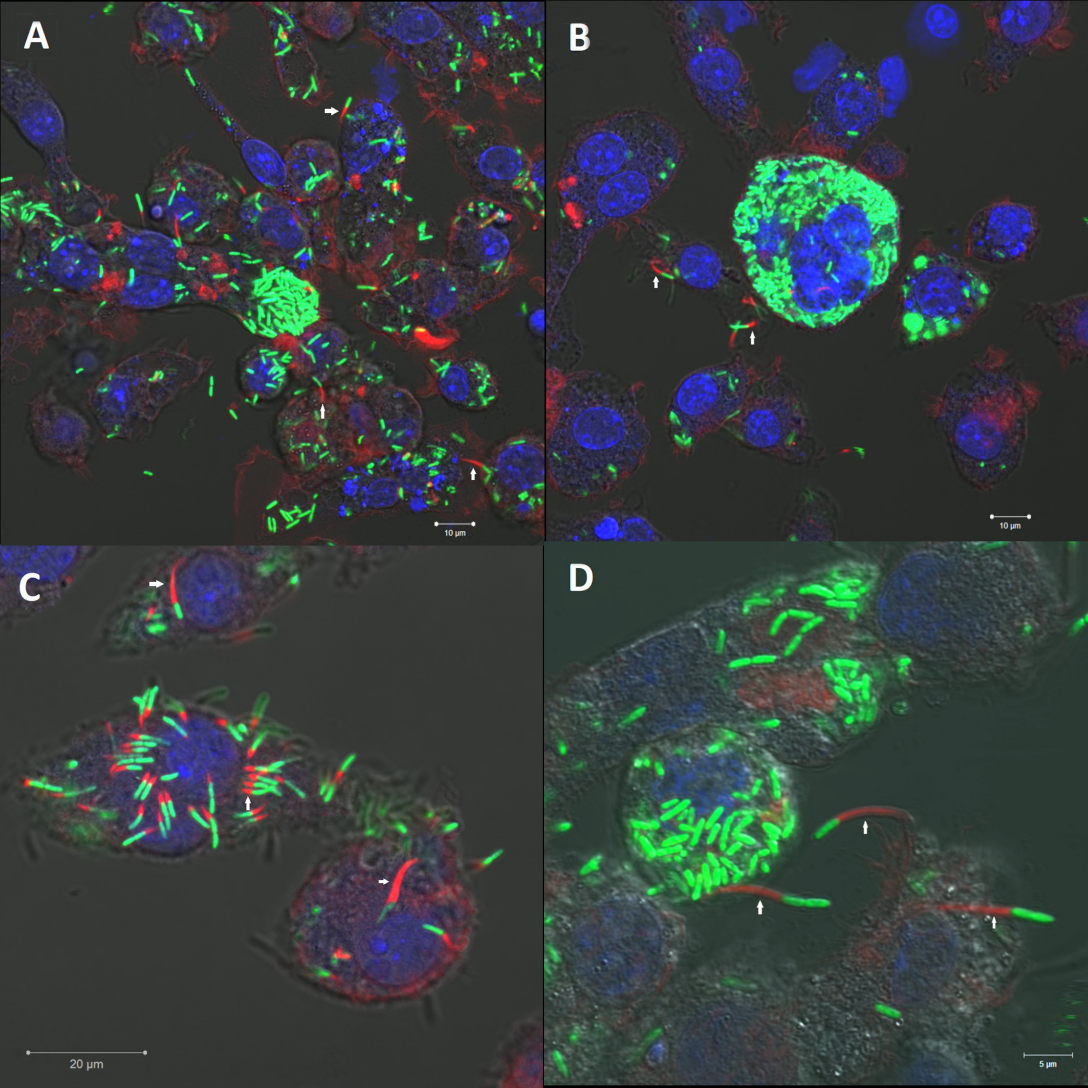
B. thailandensis E555 actin tail and multinucleated giant cell (MNGC) formation within RAW 264.7 macrophages. RAW264.7 macrophages were infected with B. thailandensis E555 enhanced green fluorescent protein (eGFP) and at 20 h postinfection were stained for actin using Alexa Fluor 647 phalloidin and a Hoechst nucleus stain. Images were taken using a confocal laser scanning microscope using 40× (A, B) and 63× (C, D) objectives. Arrows highlight examples of the bacterial actin tails.

Confocal microscopy was used to visualize infection of RAW 264.7 macrophage cells with B. thailandensis E555 enhanced green fluorescent protein (eGFP) over 24 h. Actin tail staining using fluorescently labeled phalloidin reveals actin tail formation by intracellular B. thailandensis E555 (Fig. 2).

An open-source computer program, Icy (29), was used to analyze confocal microscopy images by quantifying the intensity of GFP-B. thailandensis and the intensity of red phalloidin-stained actin tails, generating a ratio between the two.

**FIG 3:**
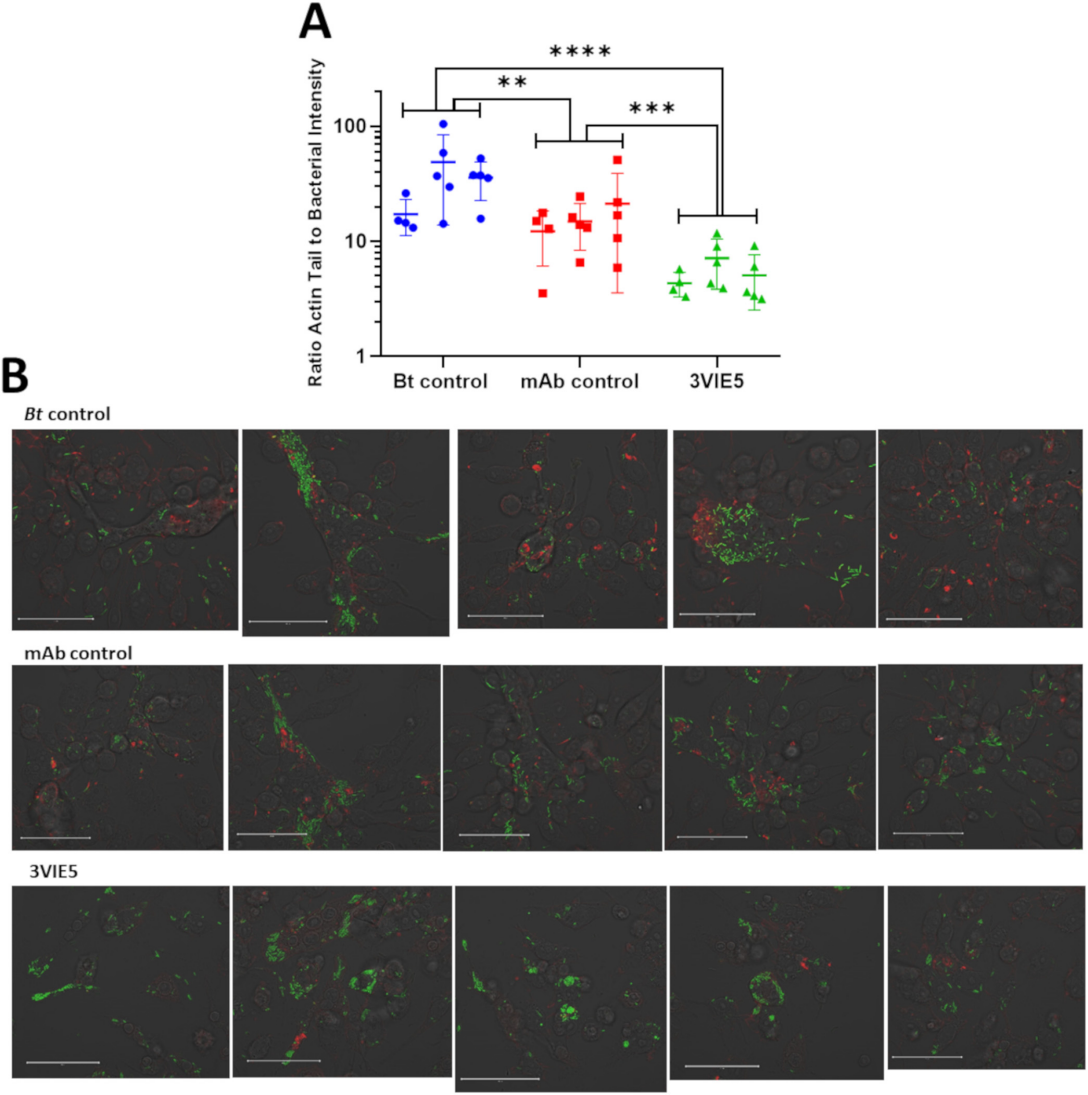
Ratio of actin tail fluorescence to bacterial fluorescence. (A) Bacterial actin tail fluorescence to GFP ratio was analyzed at 12 h postinfection using Icy open-source software. Data consist of 14 data points from 3 separate experimental weeks. **, *P* = 0.0038; ***, *P* = 0.0002; ****, *P* < 0.0001 (two-way analysis of variance [ANOVA] with Tukey’s multiple-comparison test). (B) Example images used to calculate the actin tail to GFP fluorescence ratio. These images are a single representation of all the images taken, as the original images are 8,780 by 8,780 pixels. The images shown here are the central image from which the 5-by-5 tile scan was produced. Bar, 50 µm.

Plotting the ratio generated from the GFP and phalloidin fluorescence clearly demonstrates a difference in actin tail formation between 3VIE5 MAb-opsonized B. thailandensis and nonopsonized B. thailandensis in RAW 264.7 macrophages (Fig. 3). The B. thailandensis cells opsonized with the anti-capsule MAb 3VIE5 have a reduced intensity of actin tail fluorescence compared to that of both the isotype control antibody and the nonopsonized bacterial control (*P* < 0.001 and *P* < 0.0001, respectively, using analysis of variance [ANOVA]). It is interesting that the isotype control antibody also has a reduced actin tail formation ratio compared to that of the nonopsonized control (*P* < 0.01). Our theory to explain this observation is that the isotype control antibody is causing nonspecific activation of the RAW macrophages through the interaction with their Fc receptor, thus increasing their activation status. This, in turn, leads to compartmentalization of the bacterium, thereby suppressing bacterial actin tail formation.

### Opsonization does not increase bacterial colocalization with LAMP-1-positive phagolysosomes.

The MAb 3VIE5 has demonstrated the ability to opsonize and reduce actin tail formation *in vitro*. Although it is known that B. pseudomallei and B. thailandensis are both able to escape the phagolysosome (12, 14, 15), it is not known how MAb 3VIE5 will affect the intracellular fate of the opsonized bacteria. It is possible that opsonized bacteria are unable to escape from the phagolysosome, which would be a desirable characteristic for an antibody-based therapy.

**FIG 4:**
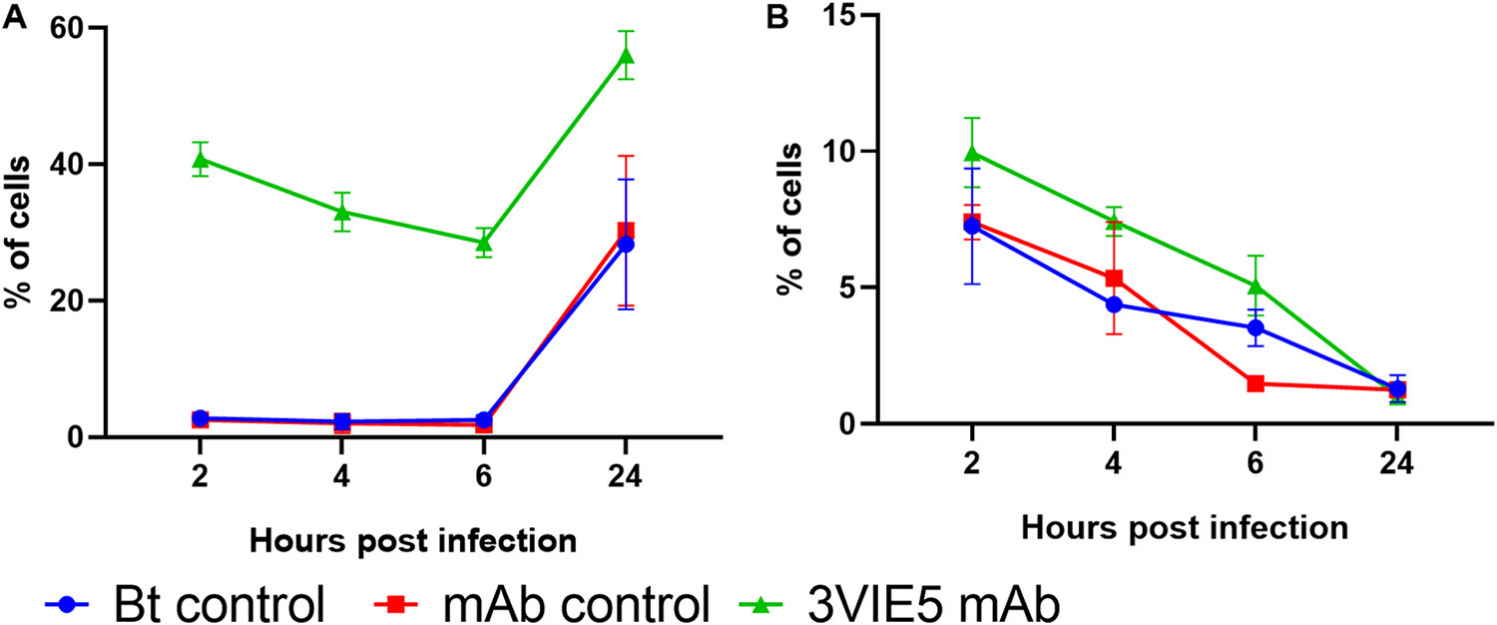
Effects of opsonization on RAW 264.7 macrophage association to B. thailandensis (A) and colocalization between intracellular B. thailandensis and LAMP-1 (B). Data shown are derived from two experiments (both derived from the mean of two biological replicates) and are shown as a repeated-measures plot. Repeated-measures ANOVA indicated a time effect in both plots (*P* < 0.001). Bonferroni’s posttests indicate a difference in uptake between the 3VIE5 MAb and the control groups (*P* < 0.05) (A), and the ANOVA test indicated possible differences in LAMP-1 (*P* < 0.05) (B); however, these differences were not discernible by posttest.

A B. thailandensis E555 GFP macrophage infection assay with imaging flow cytometry analysis was used to investigate the uptake of opsonized and nonopsonized bacteria. The MAb 3VIE5 was used in a RAW 264.7 macrophage infection assay at 1 µg · ml^−1^, which has already been shown to be opsonizing, compared to an isotype control antibody and no-antibody controls. At each time point, cells were harvested and analyzed by imaging flow cytometry; this generated a percentage of cells infected with B. thailandensis E555 GFP (Fig. 4A). Statistical analysis of these data indicated that cell association with bacteria changed over time (*P* < 0.001; repeated-measures ANOVA) and that the MAb 3VIE5 enhanced association compared to that in the two control groups (*P* = 0.022 versus no-antibody and *P* = 0.020 versus isotype control; Bonferroni’s posttests). An increase in association between cell and bacteria with MAb 3VIE5 is to be expected, since we have previously demonstrated the opsonization ability of this MAb.

It is known that binding of MAb to activating FcγRs increases the number bacteria associated with lysosomes and that this process can be independent of MAb opsonization of the bacteria (30, 31). In order to assess bacterial association with lysosomes, the lysosome marker lysosomal associated membrane protein 1 (LAMP-1) was detected with the use of a fluorescently labeled antibody and image analysis software (see Fig. S2 in the supplemental material). Statistical analysis of these data indicated that LAMP-1 association with bacteria changed over time (*P* = 0.003; repeated-measures ANOVA). We also note that there was some evidence for difference between treatment groups (*P* = 0.010; repeated-measures ANOVA); however, the effects were too modest to identify the nature of which groups might be different from each other (*P* > 0.05 in all cases). These data indicate that 3VIE5 MAb opsonization enhanced bacterial uptake; however, we could not detect a significant enhancement in maturation of the phagolysosome.

## DISCUSSION

Here, we investigated the ability of monoclonal antibodies to opsonize B. pseudomallei. A macrophage infection assay was chosen as the primary *in vitro* method to analyze intracellular infection and the effect of antibody opsonization.

Initially, B. thailandensis E555 was used in the development and testing of macrophage infection assays before progression to infection assays with B. pseudomallei K96243. B. thailandensis E555 has been shown to be a suitable surrogate for B. pseudomallei K96243 compared to other B. thailandensis strains (32). Data from J774 macrophage infection assays show similar intracellular growth profiles for B. thailandensis E555 and B. pseudomallei K96243 (32), thus confirming the utility of E555 as a surrogate for B. pseudomallei in macrophage infection studies.

The opsonization ability of the MAbs was consistent across bacterial strain and analysis technique. The consistent results between the two *Burkholderia* species tested provided the opportunity to focus our research on B. thailandensis, which can be handled at a lower level of containment, where a greater spectrum of analysis methods is available. Imaging flow cytometry combines the high-throughput aspects of standard flow cytometry (analyzing many cells though a flow cell) with the informative aspects of microscopy delivering thousands of images of cells. A B. thailandensis E555 strain expressing green fluorescent protein (GFP) was used to enable visualization of intracellular bacteria. Imaging flow cytometry was performed on RAW 264.7 macrophages harvested intact from a B. thailandensis E555 GFP infection assay to determine the opsonization ability of each MAb. The analysis of imaging flow cytometry was based upon a novel analysis and gating strategy (see Fig. S1 in the supplemental material). This strategy delivered information such as the proportion of cells infected and the relative bacterial load.

It has been demonstrated here that imaging flow cytometry is an alternative technique that can be successfully used to assess antibody opsonization within a macrophage infection assay. Imaging flow cytometry offers advantages over CFU analysis, such as the ability to analyze individual cell infection events, rather than the infection level of the whole population. It is to be noted that both methodologies have advantages and disadvantages. For example, CFU data are based on viable bacterial count on a population level, whereas imaging flow cytometry data are unable to distinguish between live and dead bacterial fluorescence. The comparable data from both methods increase the confidence in the data set. On a cell-by-cell level, antibody opsonization was shown to increase the average number of bacteria per cell, as well as increasing the overall percentage of infected cells in the population. Together, CFU and imaging flow cytometry analysis provide a robust method for analyzing intracellular infection levels in cell infection assays.

We observed that MAb opsonization reduced bacterial actin tail formation *in vitro*. It is known that B. thailandensis is able to form actin tails, similarly to B. pseudomallei and B. mallei, and that this is achieved by the conversion of G actin to F actin following bacterial escape from phagosomes into the cytosol (18, 21, 33). It is believed that bacterial actin-based motility can cause neighboring cells to contact, allowing the type VI secretory system of *Burkholderia* to cause cell fusion, leading to MNGC formation (24). The mechanism whereby the anti-capsule MAb 3VIE5 reduced actin tail formation is unknown. It is possible that MAb opsonization activates the cell, resulting in increased bacterial association with phagolysosomes and fewer cytosolic bacteria. However, in this study, no significant increase in association between bacteria and LAMP-1 was observed following antibody opsonization. Alternatively, opsonized bacteria could still be escaping from phagosomes but the MAb prevents the bacteria from recruiting and polymerizing host actin in the cytosol. The mechanism for preventing access to host actin by the MAb is unknown; it could be as simple as the MAb essentially blocking access to the actin. However, elucidating the exact mechanism requires further research.

Reducing actin tail formation *in vitro* is a desirable characteristic of a therapeutic MAb for melioidosis. The ability of the MAb to reduce actin tail formation should, in theory, reduce the spread of infection between neighboring cells. Cell fusion enables the bacteria to spread intracellularly between cells, avoiding the extracellular environment, which may contain antibiotic therapy or host immune responses, such as specific bacterial antibodies. It is known that neutrophils (34, 35) and NK and CD8 cells (36) are likely to contribute to removal of bacteria. As an antibody therapy, the anti-CPS MAb 3VIE5 could be particularly useful as a combination therapy with antibiotics, and potentially with other host-directed compounds, such as autophagy inducers (37). This could involve utilizing the MAb to prevent bacterial motility within the intracellular environment, while the secondary component, such as the autophagy inducer or antibiotic, promotes bacterial clearance. We have shown that antibody opsonization of extracellular B. thailandensis can decrease actin tail formation, which in turn could reduce intracellular bacterial spread, which is a desirable property for a potential therapeutic.

It is unclear as to whether enhanced uptake by phagocytes would be of therapeutic value. The intracellular compartment may well provide a growth niche for B. pseudomallei (discussed in the introduction). As such, enhancing uptake may seem like folly. However, by using opsonization, we are targeting uptake into immune effector cells that could be activated to kill the bacteria. It is clear, however, that an MAb therapy alone is not the complete solution to infection. We are therefore combining MAb therapy with antibiotics by developing a proof-of-concept antibody-antibiotic conjugate (AAC) for melioidosis; at the time of writing, this work is still ongoing. Antibody conjugates have the potential to enhance killing by colocalizing antibiotic to bacterium within the intracellular phagosome environment. This has been demonstrated by recent developments of an AAC for Staphylococcus aureus and Pseudomonas aeruginosa (38–41). We are currently developing an AAC for melioidosis with the MAb 3VIE5 assessed in this opsonization study. This MAb has been linked to two different antibiotics via a cathepsin-cleavable linker and is currently undergoing *in vitro* assessment. The aim of this AAC is to use the opsonization ability of 3VIE5 to deliver antibiotic intracellularly, killing B. pseudomallei bacteria residing within the intracellular environment. The development of an AAC requires the use of a well-characterized antibody with a known ability to opsonize and target the bacterium of interest. In this study, the data we have outlined lays the foundation for the development of an AAC for melioidosis.

In summary, we have shown that a panel of monoclonal antibodies, directed against the capsule and LPS of B. pseudomallei, are opsonizing in a RAW 264.7 macrophage infection assay. Furthermore, an antibody directed against the capsule of B. pseudomallei has demonstrated the ability to reduce bacterial actin tail formation within macrophage cells, an important process that the bacterium requires for intracellular motility and, ultimately, for cell-to-cell spread. Imaging flow cytometry has been demonstrated to be an alternative to the CFU method for analyzing antibody opsonization of bacteria. In addition, this method offers a greater level of detail for analyzing antibody opsonization on a single-cell level. A capsule MAb analyzed in this study, 3VIE5, is being developed into an AAC as a proof-of-principle therapeutic for melioidosis.

## MATERIALS AND METHODS

### Macrophage cell culture.

RAW 264.7 macrophages (ECCAC) were routinely cultured in Dulbecco’s modified Eagle’s medium (DMEM; Gibco) supplemented with 10% inactivated fetal bovine serum (Gibco) and 2 mM l-glutamine (Gibco). Macrophage cells were cultured within 150-cm^2^ vented cell culture flasks (Corning) within a 37°C and 5% carbon dioxide humidified incubator. Cells were regularly passaged by cell scraping and culture into fresh DMEM when approximately 90% confluent; cells were not used past a passage number of 20.

Prior to use for an infection assay, macrophages were assessed for viability and counted by trypan blue (Sigma) cell exclusion using an automated cell counter (Nexcelom). Cells were typically cultured on 24-well cell culture plates (Corning) in DMEM at a concentration of 5 × 10^5^ cells · ml^−1^ and cultured overnight within a 37°C, 5% carbon dioxide humidified incubator until cell density reached 1 × 10^6^ cells · ml^−1^.

### Macrophage infection assay.

**FIG 5:**
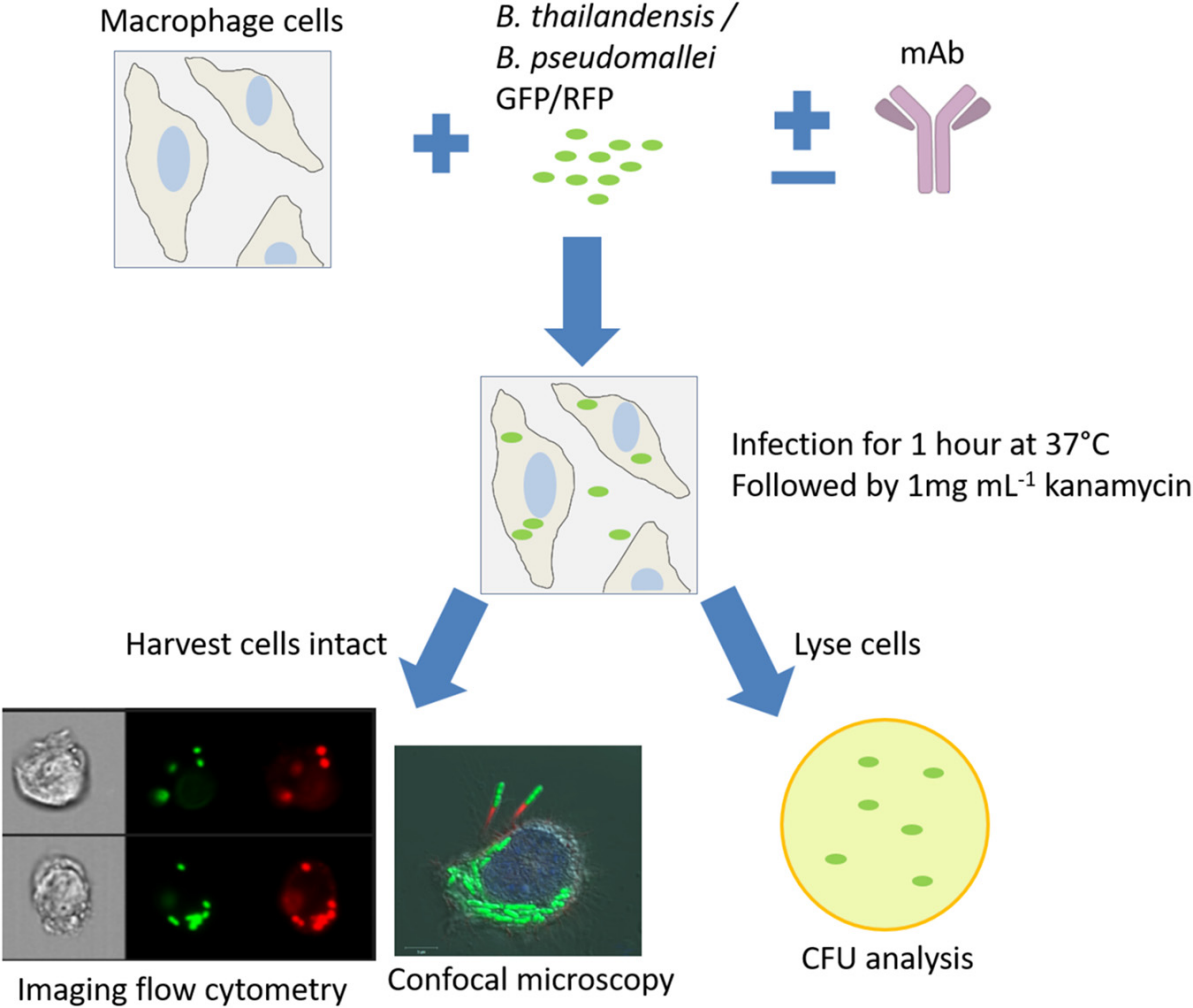
Macrophage infection assay. A RAW 264.7 macrophage infection assay was the primary method to analyze MAb opsonization of B. pseudomallei and B. thailandensis. Antibodies were incubated with bacteria prior to cell infection to allow for antibody-bacterium binding. Cells were infected for 1 h, followed by removal and replacement with kanamycin antibiotic medium. At specific time points, cells were harvested for analysis by imaging flow cytometry and confocal microscopy, or cells were lysed for bacterial enumeration.

An overnight broth culture of B. thailandensis E555 or B. pseudomallei K96243 was diluted to an approximate concentration of 1 × 10^8^ bacteria · ml^−1^ using bacterial optical density (OD) readings of 0.172 OD at 600 nm (OD_600_) and 0.4 OD_590_, respectively. A 10% dilution of this bacterial suspension was prepared and added to RAW 264.7 macrophages at a multiplicity of infection (MOI) of 5 for 1 h at 37°C to allow bacterial uptake. Following the incubation period, the culture medium was removed and replaced with fresh Leibovitz’s L-15 medium containing 1 mg · ml^−1^ kanamycin (Sigma) and incubated at 37°C to kill any remaining extracellular bacteria. This step was considered the start of time points to monitor intracellular bacteria. Following a specific incubation period (2 h for opsonization studies), the intracellular bacteria were enumerated by cell lysis with distilled water (Gibco) for a minimum of 10 min, serially diluted, plated onto Luria agar, and incubated at 37°C for 48 h. Macrophages were also harvested into phosphate-buffered saline (PBS) without lysis for analysis by imaging flow cytometry; in this case, macrophages were centrifuged at 300 × *g* for 5 min and resuspended into 4% paraformaldehyde (Alfa Aesar) prior to analysis. See Fig. 5 for an overview of the assay design.

### Culture of B. thailandensis and B. pseudomallei.

B. thailandensis E555 and B. pseudomallei K96243 were routinely cultured in Luria broth and on Luria agar plates. Green and red florescent protein-expressing strains (pBHR4-groS-eGFP and pBHR4-groS-RFP) of B. thailandensis E555 and B. pseudomallei K96243 were provided to Dstl by Exeter University, United Kingdom. Fluorescent strains were cultured in Luria broth and agar supplemented with 50 µg · ml^−1^ chloramphenicol to ensure that only plasmid-containing fluorescent protein-expressing bacteria were cultured. Culture of B. thailandensis was performed within containment level 2 facilities and culture of B. pseudomallei within containment level 3 facilities.

### Actin tail staining.

RAW 264.7 macrophages on a 35-mm cell culture dish (Corning) were fixed and permeabilized using a Cytofix/Cytoperm (BD Biosciences) kit following manufacturer’s instructions. Alexa Fluor 647 phalloidin (Molecular Probes) was added to the RAW macrophages at 5% (vol/vol) and incubated at room temperature for 30 min. Following incubation, the RAW macrophages were washed twice with PBS, prior to visualization by confocal microscopy.

### LAMP-1 staining.

At the specified time point (between 2 and 24 h) postinfection, macrophages were harvested, transferred to 200 µl fixative-permeabilization buffer (BD Biosciences) and incubated for 30 min at room temperature on a roller. Following incubation, the sample volume was increased to 1 ml with 800 µl of 1× permeabilization-wash buffer (BD Biosciences) and centrifuged for 5 min at 300 × *g*. Following centrifugation, cells were resuspended into 200 µl permeabilization-wash buffer (BD Biosciences) containing 3.5 µg · ml^−1^ PE/Dazzle 594 anti-mouse CD107a (LAMP-1) antibody (BioLegend, UK) and incubated for 1 h at room temperature on a roller. Following incubation, 800 µl of PBS (Gibco) was added to the samples and centrifuged for 5 min at 300 × *g*. Finally, all cell pellets were resuspended into 50 µl PBS (Gibco) for analysis by imaging flow cytometry.

### Confocal microscopy.

Confocal microscopy was performed on a confocal laser scanning microscope (Zeiss). RAW 264.7 macrophages were analyzed on 35-mm cell culture dishes (Corning) using a 20×, 40×, or 63× oil immersion lens. Hoechst (Sigma) nuclei dye was added to RAW 264.7 macrophage cells at 2 µg · ml^−1^ to be able to discriminate between individual cells and multinucleated giant cells. Green fluorescent protein-expressing B. thailandensis E555 (pBHR4-groS-eGFP) was used throughout confocal microscopy studies.

The bacterial actin tail fluorescence to bacterial GFP ratio was calculated using Icy open-source software (http://icy.bioimageanalysis.org/) (29). To include as many cells as possible per image, the confocal microscope at ×20 magnification was set to image a 5 by 5 square tiled image around a chosen field of view, giving a total image analysis area size of 675 µm^2^ (see Fig. S3 in the supplemental material). This process was repeated at multiple other locations on the cell culture dish and replicated across three separate weeks to generate the final data set.

### Imaging flow cytometry.

Imaging flow cytometry was performed on an Amnis ImageStream X mark II imaging flow cytometer. Cell samples were analyzed in a 50-µl volume within an Eppendorf tube. In total, 10,000 events were collected, consisting of cells gated according to being in focus and single cells. Channels 1 (bright field), 2 (GFP), 4 (RFP or LAMP-1), 6 (side scatter), and 9 (bright field) were all used at full laser power, as standard, during all analysis of B. thailandensis GFP- or B. pseudomallei RFP-infected RAW macrophages. Magnification was set at ×60 for all experiments. Compensation controls were performed on experiments consisting of more than one fluorescent marker, and the compensation matrix was then applied during data analysis. Data analysis for bacterial opsonization and LAMP-1 experiments was achieved using IDEAS (Amnis) software (see supplemental material).

### Statistical analysis.

GraphPad Prism v8 software was used in the preparation of all graphs. For statistical analysis, bacterial count data and actin ratio data were log_10_ transformed to better fit the test requirement for approximate Gaussian distribution equal variance. In all analyses, the test requirements were assessed using residual plots and quantile-quantile plots of the residuals against the normal distribution. Two-way ANOVA analysis of the actin ratio data was performed using GraphPad Prism v8, and the three experiments and three conditions were used as the two factors. Three-parameter ANCOVA analysis of the opsonization in the image stream and CFU data was performed using JASP v13.1 software. The three parameters were antibody, pathogen, and dose (log_10_ transformed and used as a continuous factor). Time course data were analyzed by repeated-measures ANOVA in which treatment and experimental run were factors. All posttests were performed using Bonferroni’s correction.
