# Supplementary material for: Monoclonal Antibodies Opsonize Burkholderia spp. and Reduce Intracellular Actin Tail Formation in a Macrophage Infection Assay
Source: J Bacteriol. 2021 Oct 12;203(21):e00244-21. doi: 10.1128/JB.00244-21 (PMC8508110; doi:10.1128/JB.00244-21)
Supplement: Supplemental file 1 — Fig. S1 to S3. Download JB.00244-21-s0001.pdf, PDF file, 0.4 MB [file jb.00244-21-s0001.pdf]

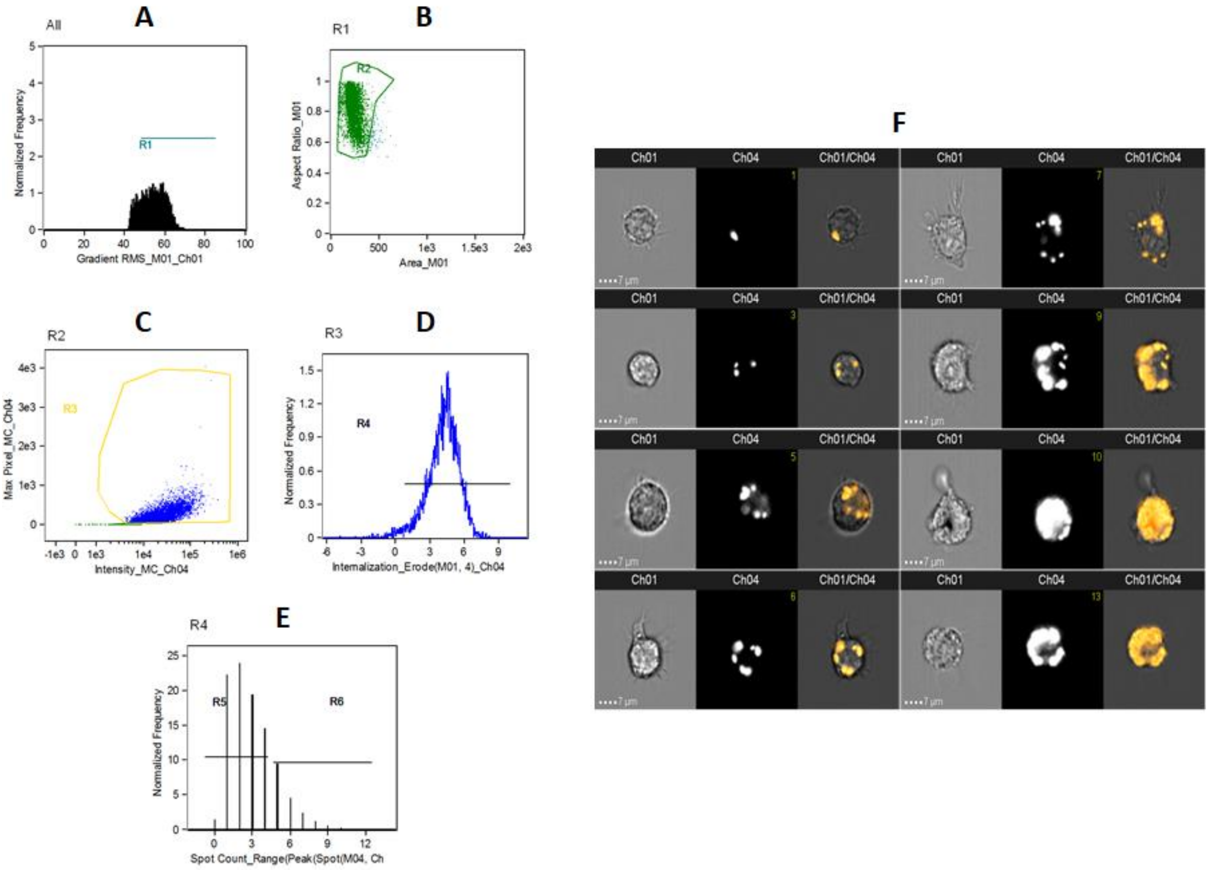

Supplemental Figure 1. Imaging flow cytometry gating strategy for monitoring intracellular *Burkholderia*.

Cells were gated based on being in focus **(A)** and single cells **(B)**. Cells associated with *B. pseudomallei* K96243 RFP were gated **(C)** and an internalisation erode applied to gate cells containing only intracellular *B. pseudomallei* K96243 RFP **(D)**. Spot count analysis **(E)** was applied to calculate high and low bacterial burden within cells. **(F)** Example images of spot counting *B. pseudomallei* K96243 RFP within RAW macrophages. The same gating strategy was applied to analysing *B. thailandensis* E555 GFP by replacing channel 4 with channel 2.

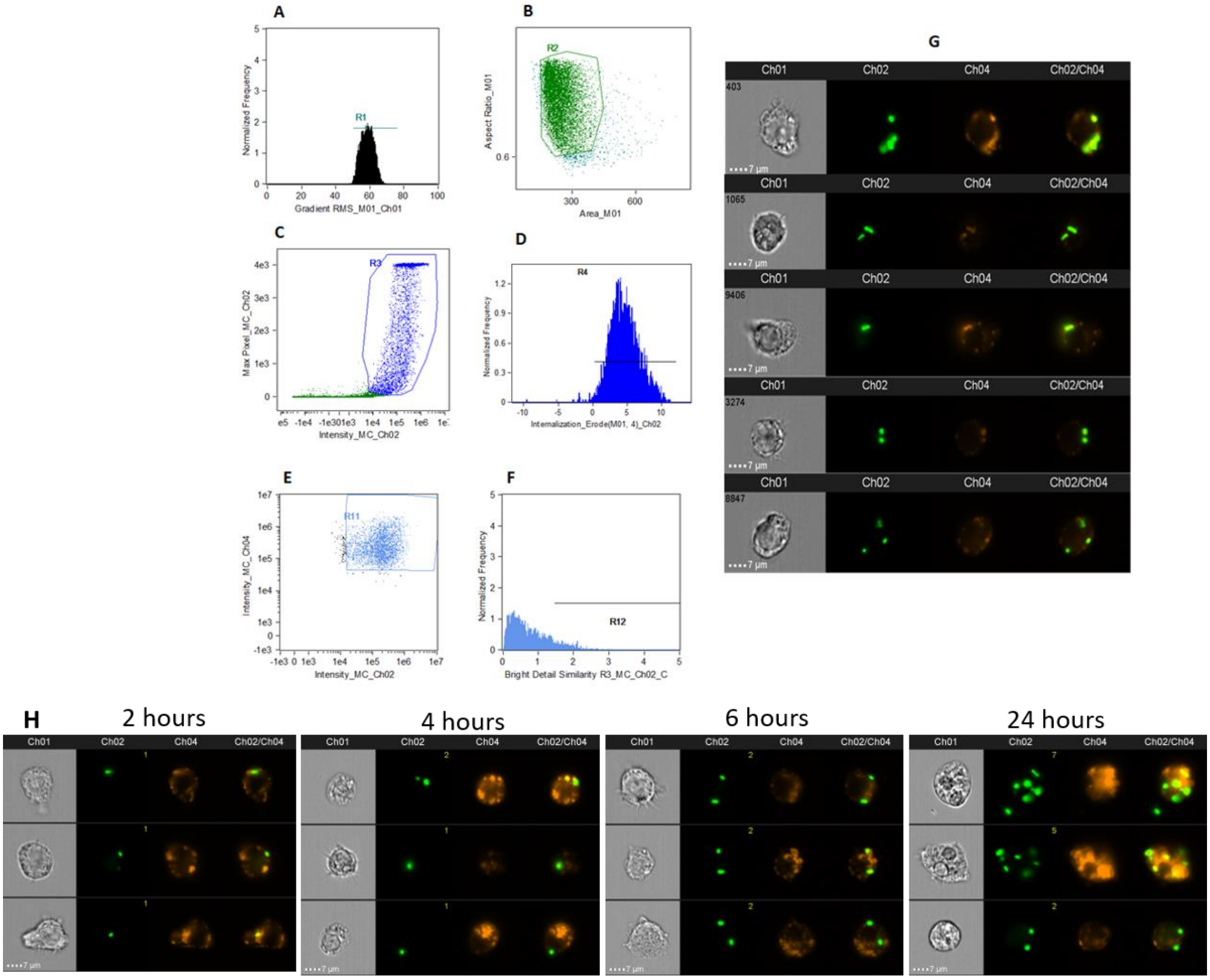

Supplemental Figure 2. Imaging flow cytometry gating strategy for analysis of GFP and LAMP-1 co-localisation.

Cells were gated based on being in focus **(A)** and single cells **(B)**. Cells associated with *B. thailandensis* E555 GFP were gated **(C)** and an internalisation erode applied to gate cells containing only intracellular GFP **(D)**. GFP and LAMP-1 (PE/Dazzle^{TM} 594) positive cells were gated **(E)** and the co-localisation of the two markers gated **(F)**. Panel **(G)** shows examples of Imaging flow cytometry GFP and LAMP-1 co-localisation. Examples of infection (GFP) and LAMP-1 at each time point (**H**), numbers present on each figure are spot counts.

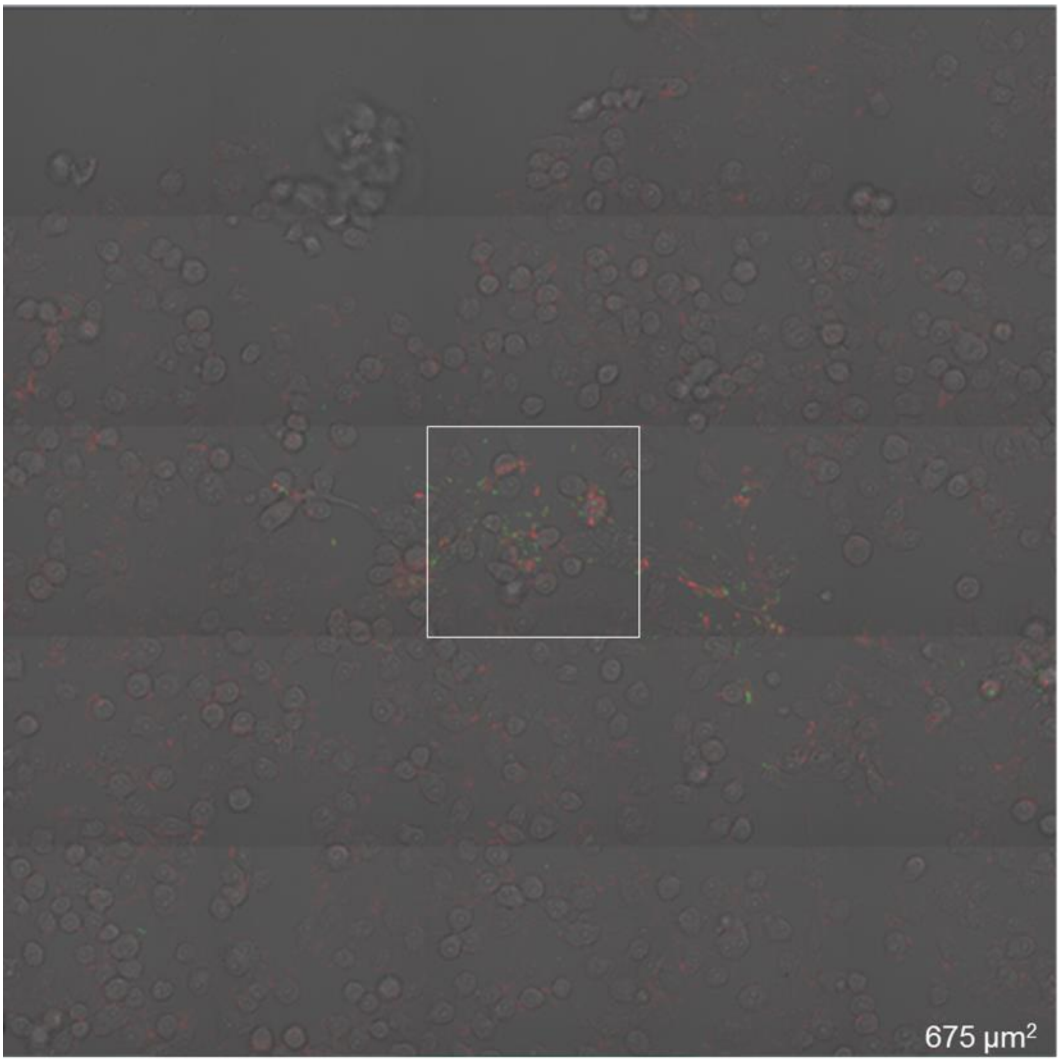

Supplemental Figure 3-. Actin tail analysis by confocal microscopy.

Confocal microscopy image of RAW 264.7 macrophage infection with *B. thailandensis* E555 GFP and actin tails stained with Alexa Fluor® 647 phalloidin. The white square represents one field of view with an x20 objective on a 35 mm cell culture dish, a 5x5 tiled image was generated around this central foci of infection to generate a total analysis area of 675 µm^2.

Images were taken using a confocal laser scanning microscope with an x20 objective and images analysed using Icy open source software.
